# Impact of Hyaluronic Acid Lip Filling on the Electromyographic Activity of the Orbicularis Oris Muscle in Adult Women

**DOI:** 10.1055/s-0045-1809151

**Published:** 2025-05-19

**Authors:** Nicole Barbosa Bettiol, Selma Siéssere, Mirella Milla Marino, Laís Valencise Magri, Jardel Francisco Mazzi-Chaves, Paulo Batista de Vasconcelos, Alice Helena de Lima Santos Cardoso, Thamyres Branco, Isabela Hallak Regalo, Simone Cecilio Hallak Regalo, Marcelo Palinkas

**Affiliations:** 1 Department of Basic and Oral Biology, Ribeirão Preto School of Dentistry, University of São Paulo, São Paulo, Brazil; 2 National Science and Technology Institute for Translational Medicine, São Paulo, Brazil; 3 Department of Restorative Dentistry, Ribeirão Preto School of Dentistry, University of São Paulo, São Paulo, Brazil

**Keywords:** hyaluronic acid, lips, orbicularis oris muscle, facial harmonization, EMG

## Abstract

**Introduction:**

The rising use of orofacial harmonization highlights the importance of the lip area, closely linked to the orbicularis oris muscle. This longitudinal study assessed the electromyographic (EMG) activity of the upper and lower orbicularis oris muscle in adult women following hyaluronic acid lip filling.

**Materials and Methods:**

Twenty-two adult women, with a mean age of 35.4 ± 12.3 years, were included. EMG was measured during rest, cheek inflation tasks (concurrently and alternately), lip protrusion and compression, and before, 30, and 60 days after lip filling. Differences were significant using *t* -test and repeated measures analysis of variance with Bonferroni correction ( *p* < 0.05).

**Results:**

Significant EMG differences were found in the upper orbicularis oris muscle (before vs. 30 days, *p* = 0.04) and lower orbicularis oris muscle (before vs. 30 days, *p* = 0.0006) during bilateral cheek inflation, and in the upper orbicularis oris muscle (30 vs. 60 days, *p* = 0.05) during rest. At 30 days, EMG of both upper and lower orbicularis oris muscle decreased in all tasks. By 60 days, the upper muscle's EMG increased in most tasks, while the lower muscle's EMG decreased during cheek inflation tasks. The lower orbicularis oris muscle had significantly higher EMG than the upper in nearly all tasks ( *p* = 0.000).

**Conclusion:**

Hyaluronic acid lip filling initially relaxed the orbicularis oris muscle at 30 days. By 60 days, the upper muscle's activity increased, indicating adaptation, while the lower muscle remained less active during cheek inflation, showing distinct functional changes after 60 days.

## Introduction

Aging affects facial harmony and symmetry due to cellular damage and genetic predispositions. 1 Over time, changes such as reduced lip volume, sagging, uneven skin tone, and bone support loss impact both function and aesthetics, influencing quality of life. 2 The lips, located at the center of the lower third of the face, are essential for facial harmony, beauty, and aesthetic appeal. This area is the most expressive in terms of movement and facial dynamics and experiences morphofunctional changes as time passes. 3 Full, well-defined lips with a reddish hue and hydration, along with elevated lip corners and a smooth texture, are associated with beauty and youth. 4

The global demand for solutions to combat aging has been increasing, leading to a notable 54.4% growth in nonsurgical aesthetic procedures over the last 4 years. 5 Among these procedures, facial fillers—particularly those based on hyaluronic acid—play a fundamental role in addressing tissue aging by restructuring and restoring volume to key areas of the face, such as the lips. As a leading nonsurgical solution for facial rejuvenation and beautification, 6 7 hyaluronic acid is naturally present in the extracellular matrix of various tissues. It is highly hydrophilic, hypoallergenic, and biocompatible, providing essential hydrodynamic properties that support hydration, tension, and tissue integrity. 8 9

Beyond its volumizing effects, hyaluronic acid may influence muscle function by altering tissue biomechanics and neuromuscular response. In the perioral region, it can modify muscle tone, elasticity, and contractile patterns, influencing facial expressions. 10 Understanding these effects is essential for optimizing aesthetic and functional outcomes, emphasizing the need for muscular assessments. 11 In this context, electromyography serves as a valuable tool for analyzing neuromuscular changes, providing deeper understanding of the functional impact of refining treatment approaches.

Moreover, this filler not only enhances lip volume but also interacts with underlying muscular structures like the orbicularis oris muscle. This muscle, consisting of superficial and deep fibers around the lips, is important for mouth-related functions, underscoring the interdependence between the structural and functional aspects of the face in the facial beautification process. 12

Despite the growing use of hyaluronic acid fillers, their functional impact on the orbicularis oris muscle remains understudied. Given its role in speech, mastication, and oral competence, filler-induced changes in tone, elasticity, and resistance could have effects beyond aesthetics. The lack of objective studies highlights the need for further research on these potential impacts.

Therefore, the objective of our longitudinal study was to evaluate the electromyographic (EMG) activity of the orbicularis oris muscle in adult women after hyaluronic acid lip filling. The null hypothesis states that no differences exist in the EMG of the orbicularis oris muscle 60 days post-lip filling.

## Materials and Methods

### Study Design and Sample Selection

This longitudinal study was approved by the ethics committee (protocol #10589419.0.0000.5419). Informed consent was obtained from all subjects.

The sample size was calculated using G*Power 3.1.9.2 (Franz Faul, Kiel University, Germany) for an a priori test (pilot project). With a significance level of *α* = 0.05, an effect size of 1.26 and a power of 84%, the calculation, based on the EMG of the upper orbicularis oris muscle during lip protrusion, determined a minimum of 10 subjects.

Among 50 women evaluated, 22 were selected based on the inclusion and exclusion criteria. Their ages ranged from 20 to 59 years (mean age 35.4 ± 12.3), normal occlusion, excluding third molars. Women exhibiting temporomandibular dysfunction, mental or physical discomfort during evaluations, mouth breathing, lip incompetence, use of muscle relaxants, speech therapy, otolaryngological treatment, recent orthodontic treatment (less than a year), or prior history of lip filling were excluded from the study.

### Hyaluronic Acid Injection

The procedure was conducted by a dental surgeon specialized in orofacial harmonization, utilizing the Restylane Kysse product, which comes in a syringe containing 20 mg/mL of hyaluronic acid (Galderma SA, Lausanne, Switzerland). Given the high mobility of this region, the use of low molecular weight hyaluronic acid was chosen to minimize interference with the natural biomechanics of lip movement. 13

The protocol followed Blandford et al's technique, 14 with the only change being the use of a 25-gauge microcannula. This method was chosen for its ease of reproducibility and ability to reduce edema and bruising, common postprocedure symptoms in the labial area after dermal fillers.

After application of operational eligibility criteria, participants underwent the following procedures before hyaluronic acid application: facial antisepsis, biosafety protocol (gown, cap, mask, and gloves), and local anesthesia, including infraorbital and mental nerve blocks. In this study, the block did not interfere with the EMG, as the assessment was conducted before the procedure and again after 30 and 60 days, ensuring accurate measurements.

**Fig. 1:**
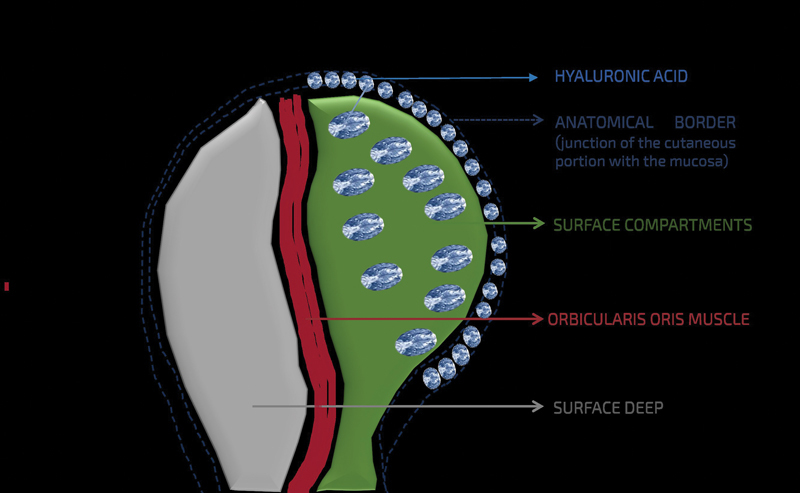
Topographic anatomy of hyaluronic acid application.

Hyaluronic acid was deposited in the superficial compartments above the orbicularis oris muscle ( Fig. 1 ). A total of 1 mL was injected, with 0.6 mL in the upper lip and 0.4 mL in the lower lip. This distribution is based on aesthetic principles and the physiological changes associated with aging. Over time, the upper lip loses more volume due to a reduction in collagen, elastin, and bone resorption, leading to thinning, elongation, and reduced vermilion exposure. Adding volume to the upper lip aims to restore its youthful proportions, balance it with the lower lip, and compensate for the loss of projection and support, resulting in a more harmonious outcome. 4

### EMG Analysis Orbicularis Oris Muscle

**Fig. 2:**
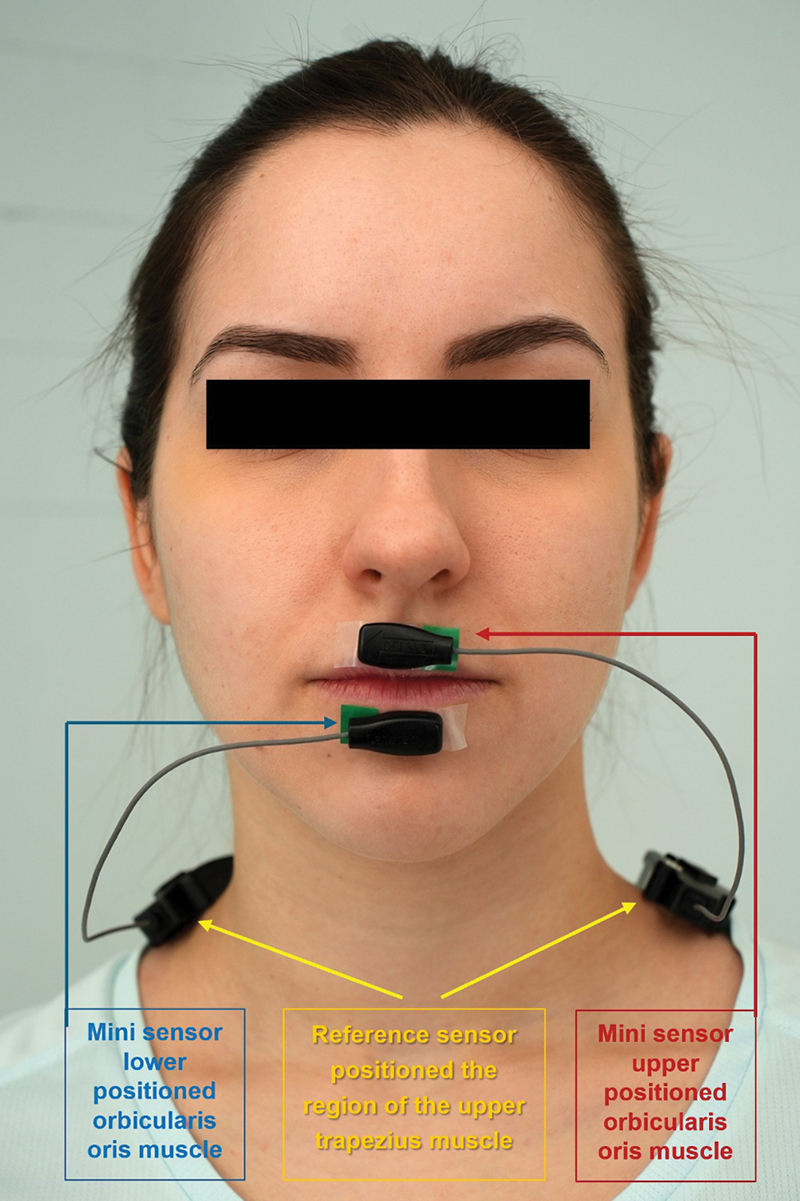
Trigno mini sensor location.

EMG recordings of the orbicularis oris muscle (upper and lower) were captured using a wireless surface EMG system (Trigno EMG sensor, Delsys Inc., United States). The wireless Trigno mini surface sensor, equipped with a smaller 25 mm EMG sensing head designed for small muscles like the orbicularis oris, was used ( Fig. 2 ).

Following the Surface EMG for Non-Invasive Assessment of Muscles Project (SENIAM) guidelines, a mini surface sensor was placed on the orbicularis oris muscle (upper and lower). 15 The reference sensor was adhered in the region of the upper trapezius muscle. Following the EMG protocol guidelines, each participant sat comfortably in a chair with hips, knees, and ankles at a 90-degree angle, maintaining an upright posture. Skin preparation involved alcohol-soaked gauze to reduce impedance and remove fat or dirt before sensor application. 16 17

EMG of the orbicularis oris muscle (upper and lower) was recorded during rest (4 seconds) and sustained isometric exercises, each lasting up to 4 seconds. These exercises included lip protrusion (pouting), lip compression (pressing the lips firmly together, activating the muscle toward the inside of the mouth), and bilateral cheek inflation (inhaling and holding air in the mouth to distend both cheeks symmetrically). Additionally, right and left cheek inflation were performed by holding air specifically on one side of the mouth. The amount of air used for cheek inflation was minimal. Tasks were repeated three times, averaging the data for final results.

The lip tasks analyzed in this study were selected for activating different portions of the orbicularis oris muscle, reflecting the motor function and physiological capabilities of this muscle. Lip protrusion assesses the activation of the fibers responsible for projecting the lips, which is essential for sound articulation and facial expressions. Lip compression activates the deep portion, important for oral control and food manipulation. Cheek inflation involves both the orbicularis oris and other facial muscles, being useful in evaluating muscle function in the context of oral dynamics.

The analysis of the EMG focused on the signal amplitude, measured in microvolts (μV), reflecting the recruitment and synchronization of motor units (motor unit action potentials). After acquiring the EMG signal data, it underwent bandpass filtering from 25 to 250 Hz and notch filtering at 50 Hz to reduce noise. Following this, the signal underwent full-wave rectification and then low-pass filtering. The evaluator, a dental surgeon, was trained in the EMG protocol.

### Statistical Analysis

The data were tested for normal distribution using the Shapiro–Wilk test. Statistical analyses were conducted with SPSS version 20.0 (SPSS Inc., Chicago, Illinois, United States). The results were analyzed using a *t* -test ( *p* < 0.05) to compare the mean EMG of the orbicularis oris muscle (upper and lower). Repeated measures analysis of variance was conducted to compare data across these time points, with Bonferroni correction ( *p* < 0.05).

## Results

Table 1 shows the EMG of the upper and lower orbicularis oris muscle before, 30 days, and 60 days after hyaluronic acid lip filling. There were significant differences in the EMG of the upper orbicularis oris muscle before versus 30 days ( *p* = 0.04) and lower orbicularis oris muscle before versus 30 days ( *p* = 0.0006) during bilateral cheek inflation after 30 days. Additionally, a decrease in EMG of the upper and lower orbicularis oris muscle was observed in all tasks at 30 days. After 60 days, the upper orbicularis oris muscle showed increased EMG in most tasks, while the lower orbicularis oris muscle showed decreased EMG in cheek inflation tasks (concurrently or alternately). Significant differences in the upper orbicularis oris muscle were also found between 30 and 60 days at rest ( *p* = 0.05).

**Table 1:** Differences in mean values (±standard error) of EMG of the orbicularis oris muscle before, 30 days, and 60 days after hyaluronic acid lip filling

EMG (µV) OOM	Periods			*p* -Value	*p* -Value (Bonferroni)		
	Before	30 d	60 d		(Before) vs. (30 d)	(Before) vs. (60 d)	(30 days) vs. (60 d)
Upper							
RE	4.06 ± 0.18	4.04 ± 0.25	4.73 ± 0.24	0.03	–	–	0.05
LP	96.95 ± 12.97	72.70 ± 9.47	87.70 ± 10.94	0.22	–	–	–
LC	97.60 ± 9.19	87.09 ± 12.98	103.85 ± 13.42	0.38	–	–	–
BCI	127.30 ± 8.21	101.52 ± 11.03	110.03 ± 11.66	0.05	0.04	–	–
RCI	120.15 ± 10.27	105.17 ± 15.53	100.88 ± 9.27	0.18	–	–	–
LCI	121.51 ± 8.68	114.49 ± 14.72	106.26 ± 10.37	0.36	–	–	–
Lower							
RE	4.98 ± 0.17	4.96 ± 0.21	5.30 ± 0.22	0.32	–	–	–
LP	148.94 ± 16.40	133.56 ± 13.31	134.41 ± 11.58	0.61			
LC	99.40 ± 10.64	83.16 ± 8.30	93.64 ± 12.90	0.45	–	–	–
BCI	178.40 ± 18.49	119.34 ± 11.91	114.92 ± 11.00	0.000	0.006	–	–
RCI	131.38 ± 14.56	109.43 ± 15.49	103.38 ± 10.67	0.08	–	–	–
LCI	126.60 ± 12.62	111.06 ± 11.59	107.31 ± 11.22	0.17	–	–	–

Abbreviations: BCI, bilateral cheek inflation; EMG, electromyographic activity; LC, lip compression; LCI, left cheek inflation; LP, lip protrusion; OOM, orbicularis oris muscle; RCI, right cheek inflation; RE, rest.

Note: Significant differences measured with repeated measures with Bonferroni correction ( *p* < 0.05).

Table 2 shows the EMG of the orbicularis oris muscle, comparing tasks between the upper and lower lips before, 30 days, and 60 days after lip filling. The lower orbicularis oris muscle had significantly higher EMG than the upper in nearly all tasks ( *p* = 0.000).

**Table 2:** Differences in mean values (±standard error) of EMG between the upper and lower orbicularis oris muscle before, 30 days, and 60 days after hyaluronic acid lip filling

Period/Task	EMG (µV) Orbicularis oris muscle		*p* -Value
	Upper	Lower	
Before			
Rest	4.06 ± 0.18	4.98 ± 0.80	0.000
Lip protrusion	96.55 ± 12.97	158.94 ± 16.40	0.000
Lip compression	97.60 ± 9.19	99.40 ± 10.64	0.000
Bilateral cheek inflation	127.30 ± 8.21	178.40 ± 18.49	0.000
Right cheek inflation	120.15 ± 10.27	131.38 ± 14.56	0.000
Left cheek inflation	121.51 ± 8.68	126.60 ± 12.62	0.000
30 days			
Rest	4.04 ± 0.25	4.96 ± 0.21	0.000
Lip protrusion	72.70 ± 9.47	133.56 ± 13.31	0.000
Lip compression	87.09 ± 12.09	83.16 ± 8.30	0.000
Bilateral cheek inflation	101.52 ± 11.03	119.34 ± 11.91	0.000
Right cheek inflation	105.17 ± 15.53	109.43 ± 15.49	0.000
Left cheek inflation	114.49 ± 15.00	111.06 ± 11.59	0.000
60 days			
Rest	4.73 ± 0.24	5.30 ± 0.22	0.000
Lip protrusion	87.70 ± 10.94	134.41 ± 11.58	0.000
Lip compression	103.35 ± 13.42	93.64 ± 12.90	0.000
Bilateral cheek inflation	110.03 ± 11.66	114.92 ± 11.00	0.000
Right cheek inflation	100.88 ± 9.27	103.38 ± 10.67	0.000
Left cheek inflation	106.26 ± 10.37	107.31 ± 11.22	0.000

Abbreviation: EMG, electromyographic activity.

Note: Significant differences, *t* -test ( *p* < 0.05).

## Discussion

The null hypothesis of this study was rejected due to significant differences in the EMG of the upper and lower orbicularis oris muscle between different time periods, after lip augmentation with hyaluronic acid.

Lip volume augmentation using hyaluronic acid can influence the function of the orbicularis oris muscle, a strong sphincter muscle linked to the dermis of the upper and lower lips via a thin, superficial muscle-aponeurotic system. The procedure can modify the soft tissue structure around the mouth, causing tissue tension or stretching and possibly affecting the muscle's natural function. Additionally, it may trigger neuromuscular adaptation, potentially impacting EMG readings. 18 19 20 21 22

EMG differences were noted in the upper orbicularis oris during rest and bilateral cheek inflation and in the lower orbicularis oris during cheek inflation. The use of hyaluronic acid on the lip may impact the EMG of the superior and inferior orbicularis oris muscle due to local inflammation. This minimally invasive procedure triggers symptoms such as redness, swelling, pain, and warmth as part of the body's natural response. 22 These inflammatory signs are essential for healing and tissue adaptation, involving the release of inflammatory mediators and increased blood flow, which supports tissue expansion. 23

Inflammation can temporarily reduce nerve excitability or impair nerve signal transmission to muscles. Muscle spindles, sensory receptors that facilitate motoneuron activity, can influence muscle function when their transduction and transmission properties are altered. 23 24 Thus, the decrease in EMG of the orbicularis oris muscle 30 days after lip augmentation is likely due to inflammation and changes in the electrophysiological and biochemical properties of the facial muscles. Hyaluronic acid aids tissue repair and regeneration by enhancing cellular signaling and modifying chemical and mechanical properties. 25

Over time, the body is expected to adapt to hyaluronic acid, leading to a reduction in symptoms. A possible explanation for the changes in the orbicularis oris muscle's EMG after 60 days could be the impact of hyaluronic acid on muscle dynamics. 26 It can alter the structure of lip tissue, affecting the alignment and function of muscle fibers. The interaction between the contractile proteins actin and myosin in the cross-bridge cycle promotes movement, but increased tissue volume and stretching can change sarcomere length and contractile efficiency, affecting the muscle's ability to generate and control force. 27 Additionally, inflammation and tissue remodeling may disrupt excitation-contraction coupling, causing temporary functional changes as the muscle adapts. 28

The impact of hyaluronic acid application on EMG signal detection is influenced by its absorption over time, which can interfere with signal capture. This interference occurs due to the increased volume conductor effect, as the geometry and conductivity of physiological tissues surrounding the sensors can alter signal conduction. For instance, the moisturizing effect of hyaluronic acid can raise the volume conductor, affecting signal capture by changing local tissue impedance and conductivity. 29 This study did not account for electrostatic factors affecting human tissues in relation to the volume conductor.

After 60 days from the lip augmentation procedure, an increase in EMG of the orbicularis oris muscle was observed in almost all tasks compared with the first month postprocedure. However, exceptions were noted during the action of inflating the cheeks to the right and left sides, where there was a gradual decrease over time.

The increase in EMG in the orbicularis oris muscle may suggest an adaptation to the structural change resulting from lip volume augmentation, leading to mucosal distension. The application of substances such as hyaluronic acid, which alters the structure of the lip mucosa, influences muscular biomechanics. These biomechanical adaptations result in slightly different lip movements, implying adjustments in tissue force that contribute to increased muscle activity. 30

However, muscle activity during the act of inflating the cheeks showed a gradual decrease over time, possibly due to neural reorganization in response to structural changes in the lips over time. So, how do we explain this decrease in muscle activity when inflating the cheeks, contrasting with the EMG of the orbicularis oris muscle in other tasks such as rest, lip protrusion, and lip compression, observed over time?

After lip augmentation, the variation in facial muscle activity can be influenced by different factors, including muscular anatomy, patterns of muscle fiber recruitment, sensory sensitivity, and neuromuscular interconnection. 31 These elements could explain the differences in muscle response between inflating the cheeks and other tasks after the lip aesthetic procedure. The orbicularis oris muscle controls the protrusion and compression of the lips, with the arrangement of fibers modulating the intensity of contraction. 32 The fibers are recruited uniformly during sustained protrusion and compression but are more localized when inflating the cheeks. 21 Sensory feedback adjusts contraction based on the perception of stretch and pressure. Coordination with other facial muscles is essential for efficient contraction and for maintaining unilateral cheek puffing, demonstrating differentiated and controlled neuromuscular coordination. 33

The results of the comparison analysis of the EMG of the upper and lower orbicularis oris muscle revealed significant differences in all tasks across the time periods. The differences in EMG between the upper and lower orbicularis oris muscles can be explained by the specific anatomy and functions of each lip region.

The lower lip plays a key role in actions like lip compression, smiling, and maintaining lip closure. These complex functions, which involve more intense muscle activity, are essential for the stability and proper functioning of the oral region. On the other hand, the upper lip performs more delicate functions such as lip pursing and fine movements, 12 which could result in lower EMG readings compared with the lower lip. Over time, neuromuscular adaptations may improve the efficiency of the lower orbicularis oris muscle, leading to higher EMG readings in that area. 30

This study had limitations. Longer longitudinal studies with extended follow-up periods are necessary for a more comprehensive evaluation of muscle adaptations over time. The age range was not controlled, and lip fillers can either enhance or limit muscle function in older individuals, depending on the technique, injection site, and rheological characteristics of hyaluronic acid. Additionally, the findings may only apply to the hyaluronic acid lip augmentation technique used and should not be generalized to other facial filling methods or materials. Finally, electrostatic properties of human tissues as a volume conductor were not considered. Furthermore, other factors that influence EMG activity, such as diet, hydration, and the menstrual cycle, were not controlled.

The study highlights the need for health care professionals to monitor and adjust lip augmentation treatments, stressing the importance of assessing changes in muscle function, in addition to facial aesthetics, within 60 days postprocedure. While further research is needed, it is essential to examine the residual effects of hyaluronic acid on muscle function before subsequent applications, especially between 6 months and 2 years after the initial treatment. These findings are vital for orofacial harmonization, helping to optimize treatment protocols for more natural aesthetic results.

Understanding facial and lip muscle dynamics can customize approaches and ensure safe, effective procedures. By linking the physiological changes caused by hyaluronic acid to the EMG activity of the orbicularis oris muscle, clinicians can predict functional adaptations, optimize injection techniques, and adjust treatment intervals to minimize adverse effects, ultimately improving patient outcomes and satisfaction by ensuring more natural results and reducing complications.

## Conclusion

This study identified distinct EMG changes in the upper and lower orbicularis oris muscles following hyaluronic acid lip augmentation. At 30 days, both muscles showed decreased EMG, indicating initial relaxation. By 60 days, the upper muscle displayed increased activity, suggesting adaptation, whereas the lower muscle remained less active during cheek inflation tasks, indicating a different pattern of adaptation. Throughout most tasks, the lower orbicularis exhibited higher EMG than the upper muscle. These results suggest that the upper muscle experienced relaxation followed by adaptation, while the lower muscle maintained reduced activity during specific tasks after 60 days. These findings suggest that clinical and functional reassessment of the orbicularis oris muscle by clinicians should be considered before touch-ups or new applications of hyaluronic acid. Neuromuscular adaptation occurs over 60 days, indicating that this period may be an appropriate interval for reinjections, minimizing interference with muscle recovery and optimizing both aesthetic and functional outcomes.
